# The role of management on costs and efficiency in HIV prevention interventions for female sex workers in Nigeria: a cluster-randomized control trial

**DOI:** 10.1186/s12962-018-0107-x

**Published:** 2018-10-23

**Authors:** S. Bautista-Arredondo, N. Nance, A. Salas-Ortiz, D. Akeju, A. G. Oluwayinka, I. Ezirim, J. Anenih, C. Chima, O. Amanze, G. Omoregie, K. Ogungbemi, S. H. Aliyu

**Affiliations:** 1 National Institute of Public Health, Mexico (INSP), Cuernavaca, Mexico; 2 University of California, Berkeley, School of Public Health (UCB), Berkeley, USA; 3 University of Lagos (UNILAG), Lagos, Nigeria; 4 Society for Family Health (SFH), Abuja, Nigeria; 5 National Agency for the Control of AIDS (NACA), Abuja, Nigeria

**Keywords:** Costs, Economic evaluation, Technical efficiency, HIV, Prevention, Community-based organizations, Management, Design thinking, Female sex workers

## Abstract

**Background:**

While the world has made much global progress toward the reduction of new HIV infections, HIV continues to be an important public health problem. In the face of constantly constrained resources, donors and grantees alike must seek to optimize resources and deliver HIV services as efficiently as possible. While there is evidence that management practices can affect efficiency, this has yet to be rigorously tested in the context of HIV service delivery.

**Methods:**

The present protocol describes the design of a cluster-randomized control trial to estimate the effect of management practices on efficiency. Specifically, we will evaluate the impact of an intervention focused on improving management practices among community-based organizations (CBOs), on the costs of HIV prevention services for female sex workers (FSW) in Nigeria. To design the intervention, we used a qualitative, design thinking-informed methodology that allowed us to understand management in its organizational context better and to develop a user-centered solution. After designing the suite of management tools, we randomly assigned 16 CBOs to the intervention group, and 15 CBOs to the control group. The intervention consisted of a comprehensive management training and a management “toolkit” to support better planning and organization of their work and better communication between CBOs and community volunteers. Both treatment and control groups received training to record data on efficiency—inputs used, and outputs produced. Both groups will be prospectively followed through to the end of the study, at which point we will compare the average unit cost per FSW served between the two groups using a quasi-experimental “difference-in-differences” (DiD) strategy. This approach identifies the effect of the intervention by examining differences between treatment and control groups, before and after the intervention thus accounting for time-constant differences between groups. Despite the rigorous randomization procedure, the small sample size and diversity in the country may still cause unobservable characteristics linked to efficiency to unbalanced between treatment and control groups at baseline. In anticipation of this possibility, using the quasi-experimental DiD approach allows any baseline differences to be “differenced out” when measuring the effect.

**Discussion:**

This study design will uniquely add to the literature around management practices by building rigorous evidence on the relationship between management skills and practices and service delivery efficiency. We expect that management will positively affect efficiency. This study will produce valuable evidence that we will disseminate to key stakeholders, including those integral to the Nigerian HIV response.

*Trial registration* This trial has been registered in Clinical Trials (NCT03371914). Registered 13 December 2018

**Electronic supplementary material:**

The online version of this article (10.1186/s12962-018-0107-x) contains supplementary material, which is available to authorized users.

## Introduction

### Background and rationale

While over 19.1 billion USD in funding was available for the HIV response in low-and middle-income countries at the end of 2016 [1], HIV continues to be an important public health problem [2]. In 2016, there were 1.8 million new infections and 1 million AIDS-related deaths globally [1]. While treatment and transmission prevention programs have gone a long way to mitigate this, access to and retention in such programs—particularly in low-and middle-income countries—remain significant barriers to end the AIDS epidemic [3]. This is particularly true for so-called key populations, such as men who have sex with men, injection drug users, and female sex workers (FSW), for whom factors like stigma and discrimination pose additional barriers to receiving prevention and treatment services.

Nigeria is a lower middle-income West African country that suffers a generalized HIV epidemic—the second largest globally [4]. As of 2016, there were 3.2 million people infected with HIV, approximately a three percent prevalence [5]. While unprotected heterosexual sex is the main mode of transmission in the country, the epidemic is particularly concentrated in high-risk populations like FSW; the prevalence among brothel-based workers is estimated to be 14.4% [6]. FSWs are specifically targeted by current national efforts for HIV prevention, including the Minimum Prevention Package Intervention, or MPPI, which seeks to reduce the burden of HIV in key populations by providing a holistic suite of behavioral, biomedical, and structural components. Specifically, the package provides testing and counseling, peer education, sexually transmitted infection (STI) treatment, and community mobilization and advocacy [7].

Given the high burden of HIV in key populations and persistent funding scarcity, strategic investment that maximizes results is a priority for governments and funding agencies [8]. Therefore, programs increasingly prioritize efficiency—broadly defined as the economic value of resources spent relative to the number of goods or services produced and expressed by the average cost per service, or unit cost. Understanding unit costs delivered (a useful measure of efficiency) and cost drivers has become an essential aspect of optimizing service delivery. Management is a determinant of productivity in manufacturing [9, 10], and is widely accepted as a key factor of success in business. Recent research has translated this insight to the health sector, finding that management can affect health service delivery and outcomes in private care settings [11]. There is a paucity of evidence, however, about the relationship between management and healthcare outcomes in non-profit settings and in low-and middle-income countries. To address this gap, the National Institute of Public Health in Mexico, the Society for Family Health (SFH), and the National Agency for the Control of AIDS (NACA) in Nigeria, have partnered to design and implement the study, “Costs, efficiency and the role of management in HIV prevention interventions for Female Sex Workers in Nigeria”.

### Study objectives and hypothesis

The present study seeks to address two principal objectives:

1. To measure the costs of implementing HIV prevention interventions and linkage to care services for FSWs in Nigeria; and
2. To experimentally evaluate the role of management practices on efficiency in community-based organizations (CBOs) in Nigeria.

We hypothesize that the intervention “package”—the data feedback, management training, and tools later described—will catalyze treatment sites to change key practices at their organizations, making service delivery more efficient.

### Trial design

This study is a cluster-randomized control trial (C-RCT) that examines the role of management practices on the costs of HIV services for FSWs in Nigeria. The project consists of two phases: (1) a design phase that uses qualitative methods to define and understand study population needs, and (2) an experimental phase, that implements a C-RCT. The design phase informed the content of a management intervention that will be implemented in CBOs and evaluated throughout the experimental phase.

## Methods

### Study setting and eligibility criteria

The study will collect and analyze efficiency data from CBOs across 14 states in Nigeria. To be included in this study, CBOs needed to (1) serve FSWs by providing HIV transmission prevention services, and (2) be funded through Society for Family Health (SFH) to offer services as part of the Global Fund or Strengthening HIV Prevention Services for Most at-Risk Populations (SHIPS for MARPs; USAID) grants. These sites offer counselling and testing, STI treatment, and HIV education services to brothels and hotspots in the surrounding area. All sites that met these criteria were included (n = 32).

### Intervention description

#### Phase I: Formative intervention design

**Fig. 1:**
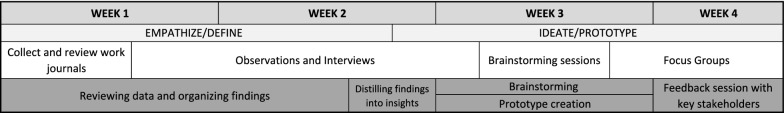
Timeline and stages of the intervention design

To inform the creation of the management intervention, we employed qualitative methods to better understand the context of the CBOs. Participants were asked to fill 24 management journals, participate in 31 key informant interviews, and 6 non-participant observations at the CBO sites. Participants included managers and volunteers from CBOs in Nasarawa and Lagos states, and Abuja (the Federal Capital Territory, FCT). The study utilized a “design thinking” approach to define organizational needs and find opportunities for management solutions. Design thinking, borrowed from engineering and now often used in business and market research, is a method that uses empathy to understand the challenges and needs of a product’s “end user” in a specific context [12]. More similar to anthropology than other more prescriptive social sciences, it focuses on the user’s feelings, moods, desires, and latent needs. The phases of design thinking with respect to our project can be divided into distinct stages in which we: (1) “empathize” with the user, (2) “define” the issue, (3) “ideate” or brainstorm solutions, and (4) “prototype” possible solutions to challenges (Fig. 1).

**Fig. 2:**
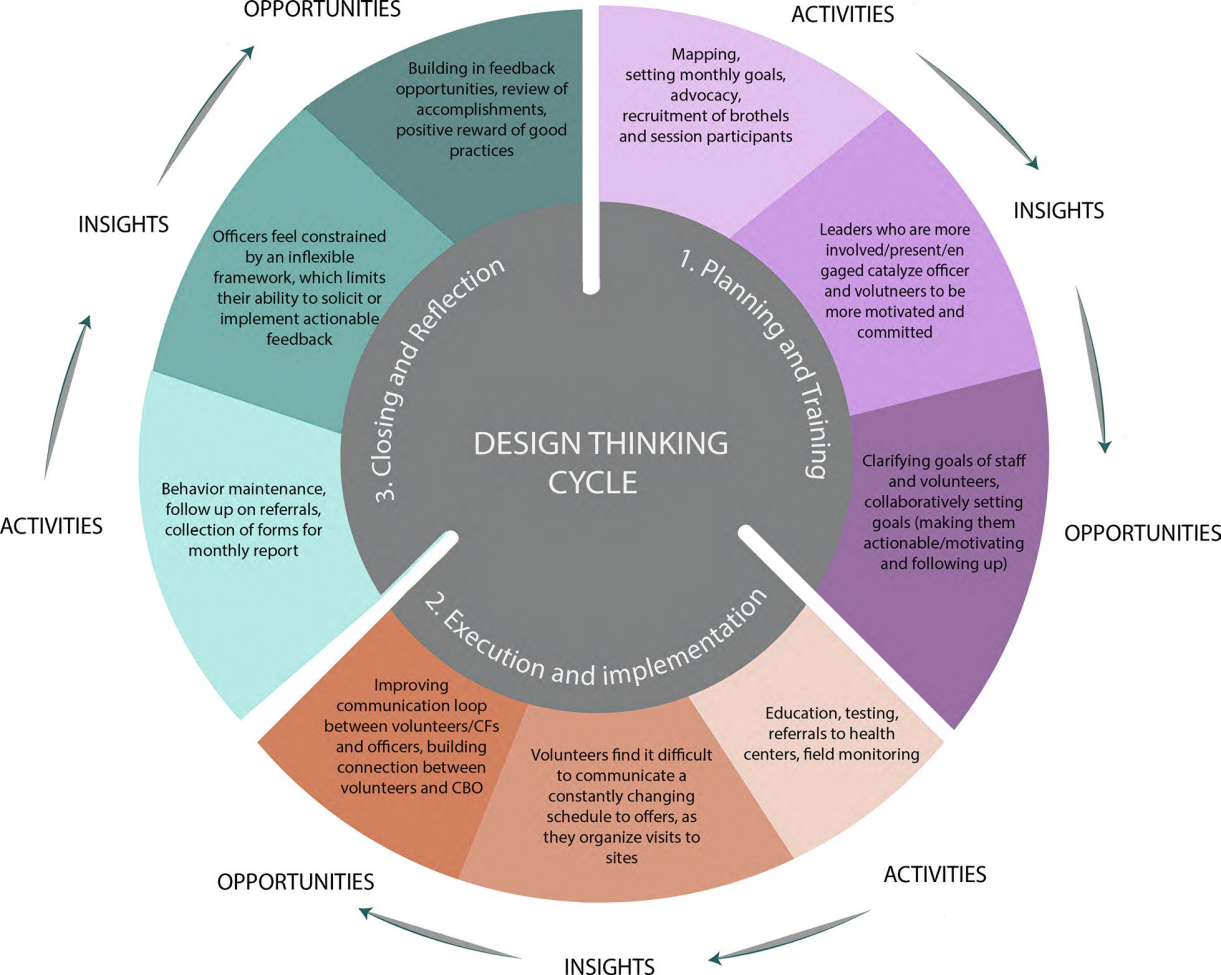
Design thinking cycle

Journals were first distributed to CBO managers and they were asked to fill these out for 3–6 weeks prior to the start of the fieldwork (see Fig. 2). Managers were to describe their daily activities and detail challenges that they encountered in their work. We then reviewed the journals before fieldwork and scheduled interviews with stakeholders, which included NACA and SFH staff, officers working at the CBOs, and volunteers providing services (11 participants had filled the journals previously). During fieldwork, we then probed for more detail about the challenges they mentioned. In all interviews, participants were asked about their work, pertinent challenges, moments of particular difficulty, and topics that elicited strongest emotions. Finally, we conducted observations, during which we spent 2–6 h passively observing the landscape and interactions at a CBO. During their time at each site, we paid particular attention to interactions between managers and volunteers, as well as the physical surroundings that could affect program implementation.

From the information gathered through journals, interviews, and observations, salient themes were found across the data—including meeting targets, funding constraints, and complications in transportation and communication. We delineated patterns, and used these to reveal “insights”. In design thinking, insights are design-ready statements that capture the latent needs or “pain points” of the user, for which we design a solution. For example, researchers found that program officers were very focused on and concerned about meeting productivity targets. While they understood the need to monitor field activities, they found the transportation and scheduling of supervision challenging, due to competing priorities. Ultimately, officers struggled to find the time to provide actionable feedback to volunteers. This insight highlights an opportunity for improvement: by building in new, realistic approaches for meaningfully communicating and interacting with volunteers, officers may be able to supervise more effectively, and volunteers may receive more useful feedback.

We then mapped the challenges, insights, and opportunities to a diagram that mirrors the program’s planning and implementation process (Fig. 2). We then used this map as a starting point to design 20+ “prototype” intervention components to address user needs. These prototypes were put to the test in three focus group sessions with stakeholders (Fig. 1). Feedback from these sessions served to finalize the management intervention. The intervention consisted of a management training, feedback loops, communication tools, goal setting, and planning tools (Fig. 2).

#### Phase II: Experimental design

##### Baseline training and data collection

Both treatment and control groups were invited to a training in Abuja, Nigeria—two managers (or “officers”) from each organization. The control group training took place on August 3rd and 4th, 2017. Trainees received one electronic tablet per CBO with Qualtrics Survey Software [13] installed. During the training, the participants were introduced to the costing component of the study, and were trained on how to collect data through the Qualtrics platform. Specifically, participants learned how to collect basic facility information, monthly inputs (condoms, test kits, etc.) used for the interventions, staff working for the interventions and their salaries, monthly rent and utilities expenses, participation in trainings, and monthly outputs produced (number of FSW served by the interventions). They subsequently collected and uploaded retrospective data for the period January 2016 to July 2017 onto the survey platform.

The treatment group training took place between August 7th and 11th, 2017, and the content was the same as that of the control group, plus an additional 3 days of management training. Topics included financial, workplace, and personnel management. The management training was broadly focused around “lean management”, a conceptual framework for improving the quality and efficiency of service delivery through small and pragmatic shifts in management practices (see Additional file 1). The attendees were also given several “management tools”—designed through the qualitative formative research—to help with their daily work. These tools included: (1) a whiteboard calendar to help them organize their constantly changing schedules, (2) “pledges” to help clarify the role of each volunteer, (3) materials and support on how to solicit meaningful feedback from volunteers, and (4) phones and phone credit to allow staff to communicate more often and effectively with volunteers in the field. As with the control group, the treatment group collected baseline data from their CBO records dated between January 2016 to July 2017.

##### Prospective intervention and data collection

Throughout the course of the study, the treatment group will receive: (1) the aforementioned “management tools” (calendar, pledges, feedback loops, and phones); (2) additional monthly “follow up” trainings via an online platform; and (3) monthly reports about their “performance”, (i.e. the data they send about inputs, outputs, and costs) (Additional file 2). The data reports will include information regarding their monthly average costs and number of FSWs reached per intervention, as well as comparisons of their performance relative to their (anonymized) peers. The online platform will provide a space through which the participants can posts successes, challenges, and solutions. This report will allow them not only to observe their performance in a specific period of time, but also to analyze changes in these outcomes over time.

### Outcomes

Our primary outcome of interest is service delivery efficiency, as measured by the CBO-level average cost per service delivered, which is referred to as *unit cost*. Unit cost will be calculated for three services provided by the CBOs: (1) HIV testing and counselling, (2) HIV education, and (3) syndromic management of STIs.

#### Unit cost

Our goal in the project is to estimate the unit costs of three prevention interventions. In order to accomplish this, we will: (1) estimate the CBO-level total costs as the sum of the quantities of key inputs used to produce the interventions multiplied by their prices; and (2) divide the total costs by the total outputs produced per intervention, per CBO.

**Fig. 3:**
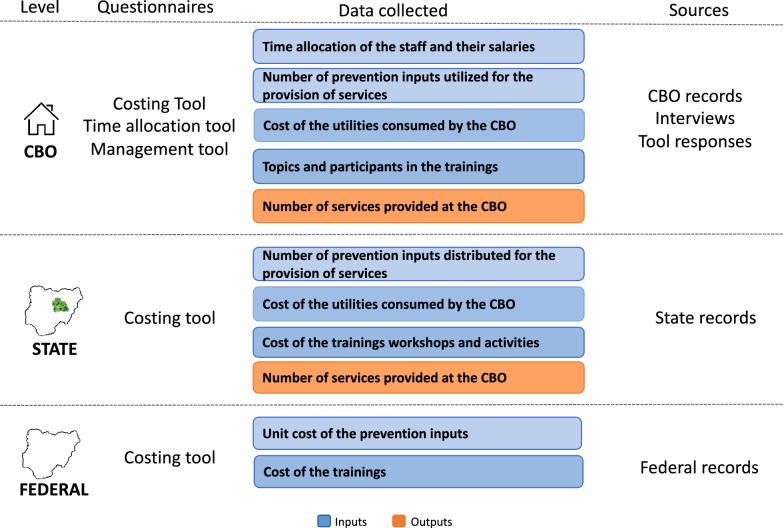
Summary of measurement methods

Inputs include staff salaries, supplies, rent, utilities, and trainings (Fig. 3). Supplies include condoms, lubricants, test kits, pamphlets, gift items or incentives. Prices will be collected from centralized records at the SFH headquarters office, and include prices of the various commodities the CBOs receive, staff salaries or payments by staff position or title, and information on trainings provided to the CBOs. Outputs include number of FSW tested, number of FSW tested positive, number of FSW educated, and number of FSW referred for STI treatment.

First, total cost will be estimated for each intervention for 2016 by summing the products of each inputs category by their prices, as follows:

1 $$TC_{ci} = \mathop \sum \limits_{j = 1}^{j = 6} (s_{ijc} *P_{j} ) + \mathop \sum \limits_{k = 1}^{k = 4} W_{i} (l_{ikc} *S_{kc} ) + \mathop \sum \limits_{m = 1}^{m = 4} (vol_{imc} *S_{mc} *Srv) + \sum {W_{i} U_{ic} } + \sum {W_{i} T_{ic} }$$

where TC_ci_ = Total costs of CBO c and intervention i, c = CBOs (n = 31), i = intervention, either 1. STI treatment, 2. HIV testing and counselling, or 3. HIV education, s_ij_ = number of supplies for each intervention i, supplies_ij_ = number of supplies for each intervention i1. 2. 3., j = supply category, 1. condoms, 2. lubricants, 3. test kits, 4. female condoms, 5. lubricants, 6. gift items (including toothbrush, toothpaste, playing cards, soap, toilet paper, etc.), P_j_ = Price of each supply j, l_ik_ = number of staff working on intervention i, by staff category k, k = staff category, 1. Executive director, 2. program officer, 3. monitoring and evaluation officer, 4. treatment program officer, etc. (varies slightly depending on program), S_k_ = annual salary paid to staff category k, vol_im_ = number of volunteers working on intervention i, by type of volunteer staff m, m = type of volunteer staff, including 1. peer educator, 2. counselor tester, and 3. venue outreach staff, 4. community facilitator (varies slightly depending on program), S_m_ = salary paid per type of volunteer staff services per service delivered, Srv = annual number of services provided by volunteer staff, W_i_ = Intervention weight, Ui_c_= Utilities costs, T_ic_ = Costs of training activities.

Since utilities and training activities are shared by all the facilities’ interventions, they will be allocated based on the proportion of clients per intervention (FSW) over the total number of CBO clients recorded for the same year (W), using the following formula:

$$W_{ci} = \frac{{FSWc_{i} }}{{\mathop \sum \nolimits_{i = 1}^{3} FSW_{i} }} ;\;W \in \left( {0,{ 1}} \right].$$

Staff time is allocated to specific interventions depending on the type of staff. Volunteer staff mostly work on a single intervention, and so their full time will be allocated to their corresponding intervention. In contrast, managerial staff work on various interventions, and their time will thus be allocated applying the same weight as seen above.

In order to estimate the average annual cost per client or unit cost (UC), the total costs per intervention will then be divided by the total number of clients served by the same intervention (NC):

$${\text{UC}}_{\text{ci}} = \frac{{TC_{i} }}{{FSW_{ci} }}.$$

#### Management practices

baseline information was collected on management practices through a survey of five-point Likert scale questions related to seven management dimensions. In total, the tool contained 82 questions. Better practices were associated with higher scores. This questionnaire consisted of different facets of the management process including: workplace organization, financial management, personnel management, supervision, transparency, accountability and community involvement. For each section of the questionnaire, one score was calculated by summing each of the values of selected options, which ranged from 0 to 5. The overall management score was then calculated as the mean score of the different management facets. In the future, the same questionnaire will be applied in two points during the implementation of the intervention—one in month six and at the end of the implementation in month 12.

### Participant timeline

**Fig. 4:**
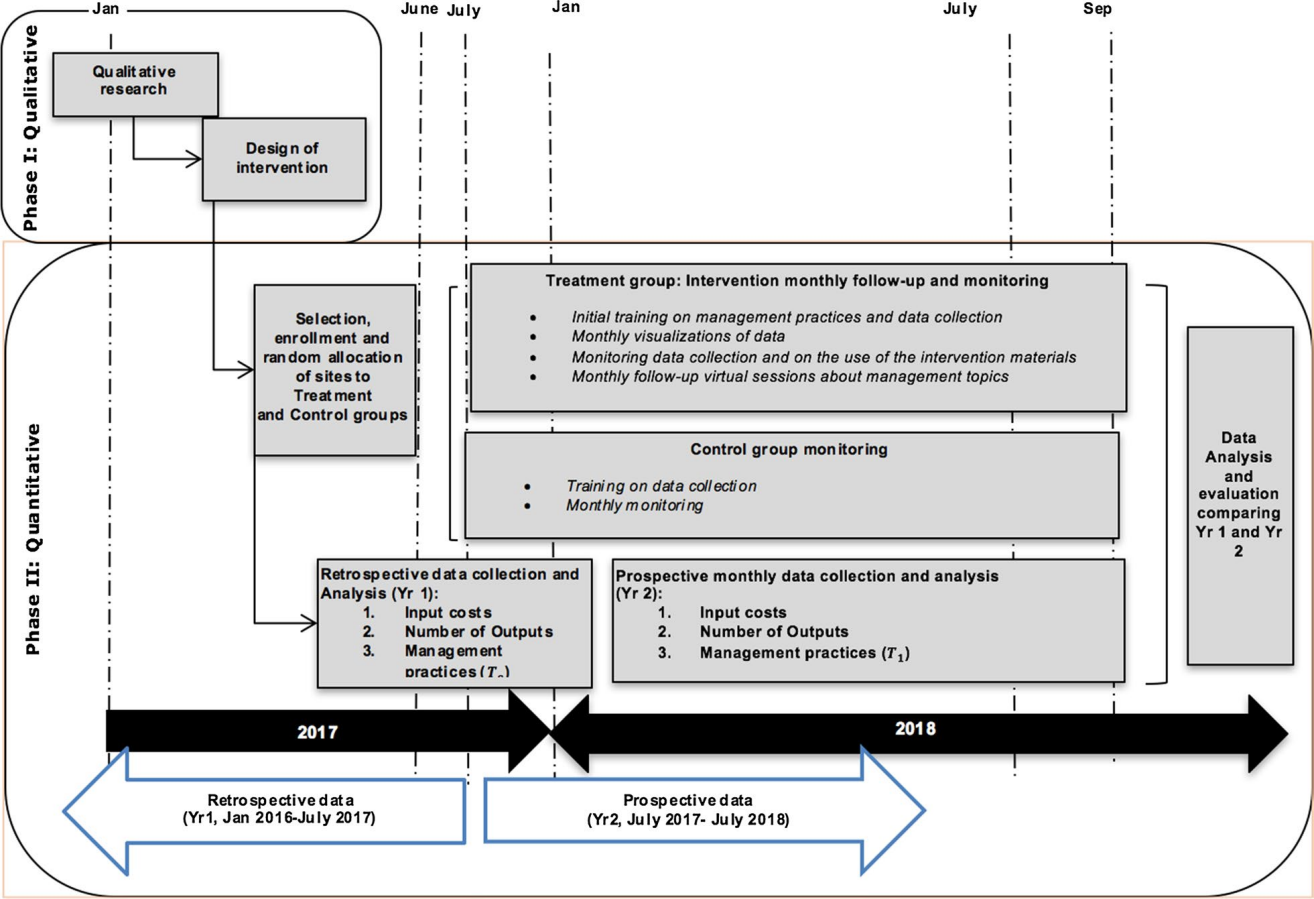
Study design and timeline

All details about the project timeline and duration of the intervention are depicted in Fig. 4.

### Sample size

Since we had a fixed number of sites (n = 32), we ran power calculations to examine the varying amounts of power that were achievable, given a variety of effect differences between treatment and control groups. The point estimate for log cost per client tested positive and accompanying standard error used in the calculations are based on data from a relevant previous costing study (ORPHEA: Assessing cost and technical efficiency of HIV prevention interventions in sub-Saharan Africa) [14–16]. Given sample size and assumptions, there was 80% power to detect a 1.09 unit difference (USD) in the log cost between the treatment and control groups.

### Allocation

**Fig. 5:**
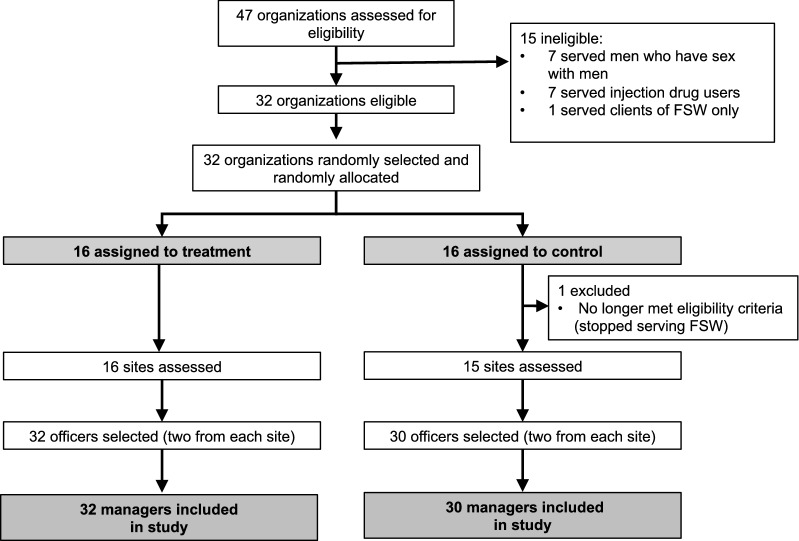
Sampling strategy

Thirty-two sites were stratified in two strata of geographic region—north and south—with 8 sites in the north stratum and 22 sites in the south stratum. The north and south regions are climatically, linguistically and culturally distinct. More importantly, the HIV epidemic also affects the regions differently; the largest concentration of HIV/AIDS is in the south. Given that such factors are likely not independent of HIV service delivery and efficiency, stratifying the randomization by these strata improves the balance between treatment groups. Within strata, we randomly assigned sites to either treatment or control groups. Treatment status was assigned using the randomization package in STATA [17]. We specified that the package run 10,000 iterations and selected the randomization with the highest (i.e. least significant) p-values across t-tests on four key input and output indicators (clients served, referrals given, condoms distributed, and number of FSW tested). We allocated 16 sites to the treatment group and 16 to the control group. One site was later excluded because it no longer served the FSW population at the start of the trial period. The final sample included 31 sites, with 16 in the treatment group and 15 in the control group (Fig. 5). We explained to participants that they were involved in a costing study, but we never revealed whether they were part of the treatment or the control groups.

### Data collection methods

Data will be collected both retrospectively (for the baseline measurement) and prospectively, from three different sources: the CBOs, the SFH state-level offices, and the SFH headquarters (Fig. 3).

For retrospective data collection, CBO managers from both treatment and control groups were asked to bring all CBO records with them to the Abuja training, and to utilize their records to input data on staff salaries, supplies, clients served, rent, utilities, and trainings at their CBO for records dated between January 2016 to July 2017. Questionnaires were built from those used previously in the ORPHEA study [16]. All participants were also asked to complete a questionnaire on management practices and job satisfaction.

For prospective data collection, training participants will collect data from their own CBO on monthly inputs (salaries, commodities, rent, utilities, and trainings), outputs (FSW served, by intervention) and management practices on their tablet with the installed survey software. Each month, participants will do this independently, but research assistants and the field data manager will review the data remotely and ask for clarification from the sites as needed. Additionally, research assistants will conduct in-person monitoring at the beginning of the prospective data collection period. The purpose of the monitoring visits will be to correct data issues and support the CBO participants on the technical aspects of tablet use as needed.

### Data management

All questionnaires will be administered using Qualtrics Survey Software [13]. Data will be downloaded as csv files and imported into STATA [17] for cleaning and analysis.

### Statistical methods

At the time of writing, baseline data cleaning was in process. Findings from both the baseline data and prospective data collection will be published in subsequent papers. For the purposes of baseline analysis, the data will be restricted to the 2016 fiscal year (January to December).

For the endline analysis, we will first assess baseline balance between treatment and control groups on key process and outcome measures, including inputs, outputs, costs and cost per FSW served. We will also examine descriptive statistics of the management scores. We will calculate unit costs and then measure the mean cost per client served at baseline and at endline for treatment and control groups. We will compare the two through multivariate ordinary least squares (OLS) regression analysis, using a “difference-in differences” (DiD) approach [18] to account for time-invariant differences between treatment and control groups:

2 $${\hbox{Y}}_{\text{ic}} = {\hbox{a}} + {\hbox{b}}_{ 1} {\hbox{x}}_{ 1} + {\hbox{b}}_{ 2} {\hbox{x}}_{2} + {\hbox{b}}_{ 3} ({\hbox{x}}_{1*} {\hbox{x}}_{2}) + [{\hbox{b}}_{4} {\hbox{x}}_{4} + \ldots {\hbox{b}}_{\text{n}} {\hbox{x}}_{\text{n}} ] + {\hbox{e}}$$

where the outcome Y is the mean cost per client served for each intervention *i*, in CBO *c* x_1_ is the treatment assignment (x_1_ = 1 if treatment and x_1_ = 0 if control); x_2_ is the time point (x_2_ = 0 if before and x_2_ = 1 if after the intervention); x_4 _− x_n_ are all variables that were identified as imbalanced at baseline. Despite the rigorous randomization procedure, the small sample size and diversity in the country may still cause unobservable characteristics linked to the implementation of services—and more importantly, to efficiency—to be unbalanced between treatment and control groups. In anticipation of this possibility, using a quasi-experimental DiD approach allows any baseline differences to be “differenced out” when measuring the effect.

## Discussion

Our study will add to the literature around management practices by building rigorous evidence on the relationship between management and service delivery efficiency. We expect that management will positively affect efficiency. As with any study, however, it is not without its limitations. The small sample size is likely to lead to wide standard errors, and the bias toward brothel-based FSW services may make the results less generalizable to non-brothel based FSW interventions.

Notwithstanding, this study will produce valuable evidence that will be disseminated to key stakeholders, including implementing partners and other actors who are key in the Nigerian HIV response. This necessarily includes our implementing partners, Society for Family Health and the National Agency for the Control of AIDS (NACA). Cost estimates will be used nationally for planning purposes. We expect that results from the randomized evaluation will inform the design of future studies examining the role of management in service delivery efficiency.

## Additional files

**Additional file 1.** Management training agenda.

**Additional file 2.** Example of baseline data report.

## References

[1] Fact sheet—Latest statistics on the status of the AIDS epidemic. http://www.unaids.org/en/resources/fact-sheet. Accessed 5 May 2018.

[2] Joint United Nations Program on HIV/AIDS (UNAIDS) (2014). The gap report.

[3] Joint United Nations Program on HIV/AIDS (UNAIDS) (2015). UNAIDS | 2016–2021 strategy: on the fast-track to end AIDS.

[4] HIV and AIDS in Nigeria. AVERT; 2015. https://www.avert.org/professionals/hiv-around-world/sub-saharan-africa/nigeria. Accessed 26 May 2018.

[5] UNAIDS. AIDSinfo | UNAIDS; 2016. http://aidsinfo.unaids.org/. Accessed 30 Apr 2018.

[6] Nigeria | UNAIDS. UNAIDS Country Statistics—Nigeria. http://www.unaids.org/en/regionscountries/countries/nigeria. Accessed 5 May 2018.

[7] National Agency for the Control of AIDS (NACA). National guidelines for implementation of HIV prevention programs for female sex workers in Nigeria; 2014. http://transglobalactivism.org/library/national-guidelines-for-implementation-of-hiv-prevention-programmes-for-female-sex-workers-in-nigeria/. Accessed 20 May 2018.

[8] Schwartlander B, Stover J, Hallett T, Atun R, Avila C, Gouws E (2011). Towards an improved investment approach for an effective response to HIV/AIDS. Lancet.

[9] Bloom N, Reenen JV (2007). Measuring and explaining management practices across firms and countries. Q J Econ..

[10] Bloom N, Eifert B, Mahajan A, McKenzie D, Roberts J (2011). Does management matter? Evidence from India.

[11] Bloom N, Sadun R, Van Reenen J. Does Management Matter in Healthcare. Lond Sch Econ Work Pap; 2013. https://people.stanford.edu/nbloom/sites/default/files/dmm_health.pdf. Accessed 20 Jan 2017.

[12] Brown T, Wyatt J (2010). Design thinking for social innovation. Dev Outreach.

[13] Qualtrics Survey Software. Provo, Utah, USA: Qualtrics Labs, Inc.; 2016. http://www.qualtrics.com. Accessed 12 Sep 2016.

[14] Bautista-Arredondo S, Sosa-Rubí SG, Opuni M, Contreras-Loya D, Kwan A, Chaumont C (2016). Costs along the service cascades for HIV testing and counselling and prevention of mother-to-child transmission. AIDS Lond Engl.

[15] Bautista-Arredondo S, Colchero MA, Amanze OO, Hera-Fuentes GL, Silverman-Retana O, Contreras-Loya D (2018). Explaining the heterogeneity in average costs per HIV/AIDS patient in Nigeria: the role of supply-side and service delivery characteristics. PLoS ONE.

[16] Bautista-Arredondo S, Sosa-Rubi SG, Opuni M, Kwan A, Chaumont C, Coetzee J (2014). Assessing cost and technical efficiency of HIV prevention interventions in sub-Saharan Africa: the ORPHEA study design and methods. BMC Health Serv Res.

[17] StataCorp (2013). STATA statistical software: release.

[18] Gertler PJ, Martinez S, Premand P, Rawlings LB, Vermeersch CM (2010). Impact evaluation in practice.

