# Supplementary material for: The role of management on costs and efficiency in HIV prevention interventions for female sex workers in Nigeria: a cluster-randomized control trial
Source: Cost Eff Resour Alloc. 2018 Oct 23;16:37. doi: 10.1186/s12962-018-0107-x (PMC6199740; doi:10.1186/s12962-018-0107-x)
Supplement: Supplementary file 1 — Additional file 1. Management training agenda. [file 12962_2018_107_MOESM1_ESM.docx]

**
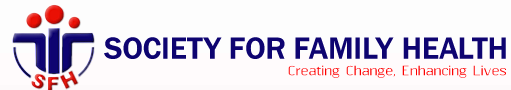
**
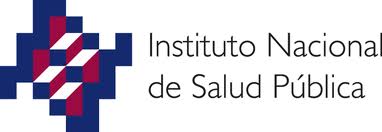

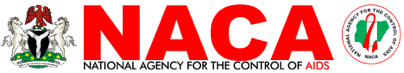


**“Costs, efficiency and the role of management in HIV prevention interventions for Female Sex Workers in Nigeria”**

# **Management Training Workshop**

August 7^th^ to 11^th^

Abuja, Nigeria

# **Total training hours:** 30

1. ***Information about the workshop***

This workshop is part of the study “Costs, efficiency and the role of management in HIV prevention interventions for Female Sex Workers in Nigeria”. The project seeks to evaluate the effect of management practices on HIV service delivery efficiency among female sex workers in Nigeria.

Topics specifically include workplace organization, financial management, personnel management and ‘lean’ management practices.

|  | **Complete Name** |
| --- | --- |
| INSP | Sergio Bautista Arredondo |
|  | Nerissa Nance |
|  | Andrea Salas Ortiz |
|  | David Akeju |
| NACA | Ezirim Idoteyin |
|  | Adejumoke Gloria Oluwayinka |
|  | Joy Egwuonwul |
|  | ThankGod Zachariah |
|  | Idepefo Festus |
|  | Anita Chinonso |
| SFH | TBD |

1. **Objectives of the workshop**

- To present the “Costs, efficiency and the role of management in HIV prevention interventions for FSWs” study to the CBOs
- To obtain input costs and number of services about the implementation of HIV prevention interventions and linkage services for Female Sex Workers (FSW) at the CBOs in Nigeria, as well as to administer the Management baseline questionnaire
- To present a basic definition and generate discussion about management practices
- To introduce the 5S methodology to the attendees, as well as some financial management practices
- To introduce the intervention materials through the lens of “lean management”

**4. Syllabus and Timeline**

| **Module** | **Topic** | **Dates/times** |
| --- | --- | --- |
| 1) Study presentation and data Collection | 1.1 Study overview and objectives | August 7th |
|  | 1.2 Qualtrics overview and troubleshooting |  |
|  | 1.3 Review of CBO costing and management instrument |  |
|  | 1.4 Real-time data collection | August 8th |
| 2) The basics of management | 2.1 What does management mean? | August 9th |
|  | 2.2 Introduction to lean management |  |
|  | 2.3 Performance management and goal setting |  |
| 3) Financial and workplace management | 3.1 Introduction to financial management practices | August 10th |
|  | 3.2 Introduction to 5S methodology |  |
|  | 3.3 Identifying strengths and opportunities |  |
| 4) Personnel management | 4.1 Root-cause problem solving and feedback loops | August 11th |

.

1. **Detailed program**

| **Module I** | ***Introduction to the “Costs, efficiency and the role of management in HIB prevention interventions for FSWs”*** |
| --- | --- |
| **Goals** | - - - - - To understand the relevance of the study and how their information will contribute to it         - To collect information regarding inputs, outputs and management practices |
| **Date and time** | - - - - - **August 7, 8:30-6:00pm** |
| **Presenters** | - - - - - Nerissa, Andrea, Ogbonna, Id, Adejumoke, Sergio, Godpower         - Facilitator: Festus |

| **Topic** | **Activities** | **Time** | **Presenter/Responsible Person/Organization** |
| --- | --- | --- | --- |
|  | 1. Arrival and registration of participants | 8:30-9:00am | SFH |
|  | 1. Opening Prayer | 9:00-9:05am | Volunteer |
|  | 1. Goodwill Messages | 9:05-9:10am | NACA, SFH |

| **Topic** | **Activities** | **Materials required** | **Modality** | **Time** | **Presenter/responsible**  **Person/Organization** |
| --- | --- | --- | --- | --- | --- |
| **1.1 Study overview and objectives** | 4. General introduction of research team and goals of the data collection  -Distribution and review of the training agenda  -Other miscellaneous logistics  -Introductions of CBO and manager names, locations, etc.  -Ice breaker activity with names | Name tags | In plenary | 9:10-9:40am | Various |
|  | 5. Presentation of the study  -Justification  -Objectives | Projector | In plenary | 9:40-10:30am | Sergio Bautista  INSP |
| **1.2 Qualtrics overview and troubleshooting** | 6. Introduction to the Qualtrics program and the tool | Projector  Booklet  Tablets | In plenary | 10:20-11:00am | Andrea Salas  INSP |
|  | 7 Morning Tea Break |  |  | 11:00-11:30am |  |
| **1.3 Review of CBO costing and management instrument** | 8. Presentation of the Costing tool | Projector  Booklet | In plenary | 11:30-12:10pm  12:10-12:30pm | Nerissa Nance  INSP |
| **1.4 Real-time data collection** | 9. Fill “checklist” of needed material and begin data collection | Tablets |  | 12:30-2:30pm | Nerissa Nance  INSP |
|  | 10. Lunch Break |  |  | 2:30-3:30pm |  |

|  | 11. Management survey  12. Continue data collection | Projector  Booklet  Tablets |  | 3:30-5:45pm | N/A |
| --- | --- | --- | --- | --- | --- |
|  | 13. Evening Tea and closing prayer |  |  | 5:45-6:00pm | SFH |

| **Module I** | ***Data collection, continued*** |
| --- | --- |
| **Date and time** | - - - - - **August 8th, 8:30-6:00pm** |
| **Presenters** | - - - - - Nerissa, Andrea, Ogbonna, Id, Adejumoke |

| **Topic** | **Activities** | **Time** | **Presenter/Responsible Person/Organization** |
| --- | --- | --- | --- |
|  | 1. Arrival and registration of participants | 8:30-9:00am | SFH |
|  | 1. Opening Prayer | 9:00-9:05am | Volunteer |
|  | 1. Goodwill Messages | 9:05-9:10am | NACA, SFH |

| **Topic** | **Activities** | **Materials required** | **Time** | **Presenter** |
| --- | --- | --- | --- | --- |
| **1.4 Real-time data collection** | 4. Continue data collection | Tablets | 9:10-11:00am |  |
|  | 5. Morning tea break |  | 11:00-11:30am |  |
|  | 6. Continue data collection | Tablets | 11:30-2:30pm |  |
|  | 7. Lunch Break |  | 2:30-3:30pm |  |
|  | 8. Continue data collection | Tablets | 3:30-5:45pm |  |
|  | 9. Evening Tea and closing prayer |  | 5:45-6:00pm |  |

| **Module II** | ***The basics of management*** |
| --- | --- |
| **Goals** | - - - - - To ensure the understanding of management and management practices by CBO managers         - To learn about goal setting |
| **Date and time** | - - - - - **August 9th, 8:30-6:00p**m |
| **Presenters** | - - - - - Nerissa, Andrea, Ogbonna, Id, Adejumoke         - Facilitator: Joy |

| **Topic** | **Activities** | **Time** | **Presenter/Responsible Person/Organization** |
| --- | --- | --- | --- |
|  | 1. Arrival and registration of participants | 8:30-9:00am | SFH |
|  | 2. Opening Prayer | 9:00-9:05am | Volunteer |
|  | 3. Goodwill Messages | 9:05-9:10am | NACA, SFH |

| **Topic** | **Activities** | **Materials required** | **Modality** | **Time** | **Presenter/responsible**  **Person/Organization** |
| --- | --- | --- | --- | --- | --- |
|  | 1. Introductions   General introduction of research team, project, goals of the training on Management  Distribution and review of the training agenda  Other miscellaneous logistics | Name tags | In plenary | 9:10-9:40am | TBD |
| **2.1 What does management mean?** | 5. Brainstorm: ask the attendees to write in post-its all the management activities that they do/have done at the CBO and put them in a chart paper | Chart paper, post-its, pens | In plenary | 9:40-10:10am | Andrea Salas  INSP |
|  | 6. Lecture: presentation “Introduction to management”   - Definition - Management Functions - What to manage? | Projector | Lecture | 10:10-11:00am | Andrea Salas  INSP |
|  | 7. Morning Tea Break |  |  | 11:00-11:30am |  |
|  | 8. In some chart papers (one for each management function) organize the post-its done in part 1.  Contrast what the attendees consider as management  Discuss with the group about which activities are missing | Chart paper | Work group | 11:30-12:30pm | Andrea Salas  INSP |
| 2.2 Introduction to lean management | 9. Lecture: presentation “Introduction to lean”   - What is lean and how it can be used - Defining performance management in lean   Activity: reflection (workbook) | Power points, notebook | Lecture | 12:30-1:30 pm | Nerissa Nance  INSP |
| 2.3 Performance management and goal setting | 10. Lecture: presentation “Introduction to capacity management and goal setting”  Activity: reflection (workbook) | Notebook, Power points | Lecture | 1:30-2:00 pm | Nerissa Nance  INSP |
|  | 11. Activity: Integrating the calendar into daily work life  As part of the training, each CBO will receive a whiteboard calendar. Here, they will review it and do a mock week-long plan for their next week at work. They will share with their group and discuss its utility | Calendar, notebook | Work Group | 2:00-2:30 pm | Nerissa Nance  INSP |
|  | 12. Lunch Break |  |  | 2:30-3:30pm |  |
|  | 13. Activity: pledges  Take the copies of the pledges found in your folder and read through them. Talk about the roles and how to use the pledges to goal set. Refer back to the goals created in the previous module. | Power points, notebook | Lecture | 3:30-4:00 pm | Nerissa Nance  INSP |
|  | 14. Activity: Creating OKR goals for staff  Participants will pick a person on your team and role play creating goals with them, then share with another pair what the pro | Handouts, notebook | Pair | 4:00-5:10 pm | Nerissa Nance  INSP |

|  | 15. Review of lessons learned (Quiz)  16. Review of next session |  |  | 5:10-5:35pm  5:35-5:45pm | Andrea Salas  INSP |
| --- | --- | --- | --- | --- | --- |
|  | 17. Evening Tea and closing prayer |  |  | 5:45-6:00pm |  |

| **Module III** | ***Financial and workplace management*** |
| --- | --- |
| **Goals** | - To show how an organized workplace matters so as to enhance the provision of HIV services - To bring tools in order to enhance decision-making about financial processes at the CBO - To create understanding on some aspects of financial and structural management |
| **Date and time** | - - - - - **August 10, 8:30-6:00pm** |
| **Presenters** | - - - - - Andrea, Nerissa and others         - Facilitator: SFH |

| **Topic** | **Activities** | **Time** | **Presenter/Responsible Person/Organization** |
| --- | --- | --- | --- |
|  | 1. Arrival and registration of participants | 8:30-9:00am | SFH |
|  | 2. Opening Prayer | 9:00-9:05am | Volunteer |
|  | 3. Goodwill Messages | 9:05-9:10am | NACA, SFH |

| **3.1 Introduction to financial management practices** | 4. Icebreaker  In groups of 2-3 people come up with some definitions: | Paper sheets | In group | 9:10-9:40 am | Andrea Salas  INSP |
| --- | --- | --- | --- | --- | --- |
|  | 5. Lecture: presentation “introduction to financial management practices” | Projector  Workbookl | Individual | 9:40-10:30 am | Andrea Salas  INSP |
|  | 6. Activity:  In groups of 2-3 people, create a budget with the materials provided | Material: a template for them to fill in |  | 10:30-11:00 am | Andrea Salas  INSP |
|  | 7. Tea break |  |  | 11:00- 11:30 am |  |
|  | 8. Discuss by teams: What are the challenges you are currently experiencing with money management in your organization? How might you solve them? | Paper sheet | Individual | 11:30-12:00 | Andrea Salas  INSP |

| **3.2 Introduction to the 5S methodology** | 9. Lecture: presentation “Introduction to 5S methodology”   - What the method is about? - Why is it helpful? - The 5S | Projector | Lecture | 12:00-12:40 am | Andrea Salas  INSP |
| --- | --- | --- | --- | --- | --- |
|  | 10. Hands-on!  Think about how the 5s method could be applied to your CBO.  *Give the participant 5 paper sheets, one S in each sheet, so that the attendees can write down their ideas about how can they implement each S in their own facility  Present the work in a group  Discuss in group about the method | “S” Paper  sheets | In plenary, individual | 12:40-1:20 pm | Andrea Salas  INSP |
| 3.3 Identifying strengths and opportunities | 11. SWOT  Lecture: presentation “Introduction to SWOT (strengths, weaknesses, opportunities, threats)”  Workbook (Activity) | -Power Point Presentation  -Sheet paper | In pairs | 1:20-2:30 pm | Andrea Salas  INSP |

|  | 12. Lunch Break |  |  | 2:30-3:30 |  |
| --- | --- | --- | --- | --- | --- |
|  | 13. Feedback from past quiz |  |  | 3:30-4:00 pm | Andrea Salas |
|  | 14.Review of lessons learned (Quiz)  15. Review of next session |  |  | 4:00-4:30pm  4:30-5:00pm | INSP |
|  | 16. Evening Tea and closing prayer |  |  | 5:00-6:00pm |  |

| **Module IV** | ***Root-cause problem solving, feedback loops and conclusion of the workshop*** |
| --- | --- |
| **Goals** | - - - - - To show how to best organize interpersonal relationships within the project team         - To integrate intervention items into works |
| **Date and time** | - - - - - **August 11^th^, 8:30-6:00pm** |
| **Presenters** | - - - - - Nerissa and others         - Facilitator: Thankgod |

| **Topic** | **Activities** | **Time** | **Presenter/Responsible Person/organization** |
| --- | --- | --- | --- |
|  | 1. Arrival and registration of participants | 8:30-9:00am | SFH |
|  | 1. Opening Prayer | 9:00-9:05am | Volunteer |
|  | 1. Goodwill Messages | 9:05-9:10am | NACA, SFH |

| **Topic** | **Activities** | **Materials required** | **Modality** | **Time** | **Presenter** |
| --- | --- | --- | --- | --- | --- |
| 4.1 Root-cause problem solving and feedback loops | 4. Case study: the Toyota story  Presenter shows the case study of Toyota motor’s stark changes in management practices  (alternatively: make up two case studies and discuss the practices they use and the positive and negative things that come from their practices. | Case studies printed | Work group | 9:10-10:10 am | Nerissa Nance  INSP |
|  | 5. Lecture: presentation “Root cause problem solving and feedback loops” | Handouts, power point, notepad | Individual | 10:10-11:00am | Nerissa Nance  INSP |
|  | 7. Tea morning break |  |  | 11:00-11:30 am |  |

|  | 6. Activity: Quote feedback loop  Ask participants to get into groups of 5-6 and go through the motions of writing down issues from the field, sharing, and brainstorming to solve them  Handout: tips for facilitating a brainstorm | Handouts, large poster paper, markers | Pairs | 11:30-12:30pm | Nerissa Nance  INSP |
| --- | --- | --- | --- | --- | --- |
|  | 7. Activity: The 5 Why’s of problem solving  Participants will pick two issues each that they see in their work and perform the “5 why’s” exercise with a partner | Handouts, large poster paper, markers | Group | 12:30 – 1:30 pm | Nerissa Nance  INSP |
|  | 8. Activity: Phone cards  As part of this training, Managers and intermediate level will get the phone cards. In Groups of 4-5, participants will discuss creating a structure of communication at their CBO (who will contact who, and when) | Notebook | Work Group | 1:30-2:00pm | Nerissa Nance  INSP |
|  | 9. Closing  Participants will share out lessons learned and will pledge how they will change their practices at the CBO | None | Group | 2:00-2:30 pm | Nerissa Nance  INSP |
|  | 10. Lunch Break |  |  | 2:30-3:30pm |  |

|  | 11. Feedback from past quiz  12. Review of lessons learned |  | |  | 3:30-4:00pm  4:00-4:30pm | |  |
| --- | --- | --- | --- | --- | --- | --- | --- |
|  | ***Conclusion*** | | | | | | |
| **Activities** | | **Materials required** | **Modality** | | | **Time** | **Presenter** |
| 13. Review of all modules and key takeaways  Discussion:  Taking home lessons learned:   - What will they put into practice tomorrow - What will they need more support with in order to put into practice - What did they like, what could be better   14. Evening Tea and closing prayer | | None | Group | | | 4:30-5:30  5:30-6:00pm | Nerissa Nance  INSP |
