# Supplementary material for: The role of management on costs and efficiency in HIV prevention interventions for female sex workers in Nigeria: a cluster-randomized control trial
Source: Cost Eff Resour Alloc. 2018 Oct 23;16:37. doi: 10.1186/s12962-018-0107-x (PMC6199740; doi:10.1186/s12962-018-0107-x)
Supplement: Supplementary file 2 — Additional file 2. Example of baseline data report. [file 12962_2018_107_MOESM2_ESM.pdf]

Baseline Data Report

CBO # 22

HIV Testing and Counseling service costs in 2016

In August of 2017, with the help of your organization, INSP collected data about supplies, staff costs, and number of female sex workers (FSW) reached by services. We have analyzed these data and the objective of these visualizations is to give you useful information about your organization. We hope this information can help guide decisions you make about your CBO. We look forward to your feedback.

Proportion of 2016 cost by type of input

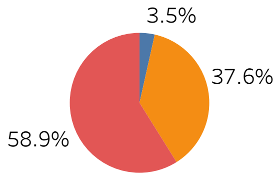

In the above chart, we can see what percent of the total cost is spent on each of the inputs. Results are only for your CBO, CBO #22.

Proportion of 2016 cost by type of input (across sites)

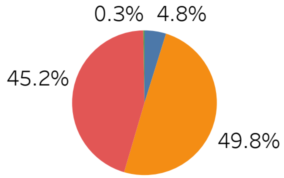

In the above chart, we can see that percent of the total cost that is spent on each of the inputs. Results are for all CBOs overall.

Input Type
- rent costs
- staff costs
- supplies costs
- training costs

For every 100 USD spent, about 50 are spent on staff, 45 on supplies, 2 on rent and utilities, and less than 1 on trainings.

Average number of FSW educated in 2016

118 — 17,578

Average number of FSW tested for HIV in 2016, by state

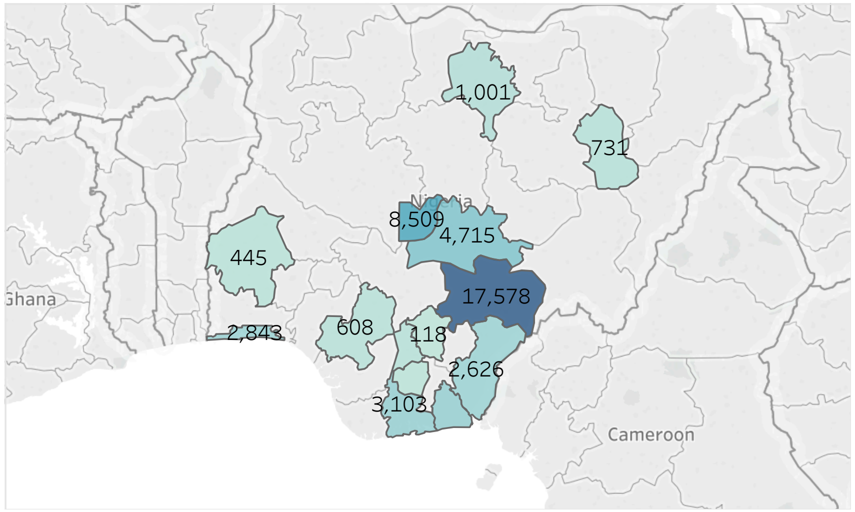

Here, by state, we see the average number of women tested each year. Darker colors mean more women tested.

Cost per FSW tested by number of FSW tested

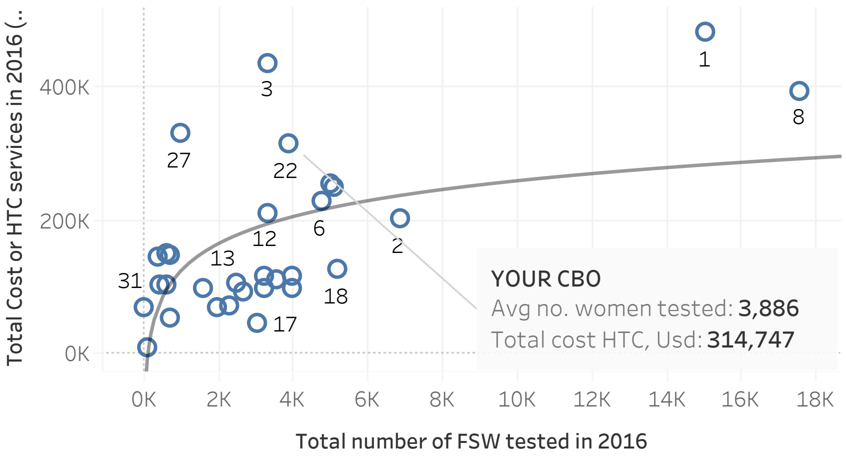

The above graph shows the total amount spent (in USD) in 2016 for HTC services, and how many women were served in 2016. In general, the more spent the more women are served. However, some sites reach the same amount of women with less money spent.
